# A new and useful tool for differentiating prolactinomas from non-functioning pituitary adenomas: a pilot study of the cabergoline disconnection test

**DOI:** 10.31744/einstein_journal/2025AO1694

**Published:** 2025-08-15

**Authors:** Stefano Alvarenga Galliano, Matheo Augusto Morandi Stumpf, Nara L. Queiroz, Eduardo Henrique Rodrigues Ferreira, Flora Ladeira Craveiro, Christiane Gruetzmacher, Gilberto Ochman da Silva, Valter Angelo Sperling Cescato, Eduardo Arnaldo Silva Vellutini, Malebranche Berardo Carneiro Cunha-Neto, Rafael Loch Batista, Andrea Glezer

**Affiliations:** 1 Universidade de São Paulo Faculdade de Medicina Hospital das Clínicas São Paulo SP Brazil Endocrinology and Metabolism Discipline, Neuroendocrinology Unit, Hospital das Clínicas, Faculdade de Medicina, Universidade de São Paulo, São Paulo, SP, Brazil.; 2 Universidade de São Paulo Faculdade de Medicina Hospital das Clínicas São Paulo SP Brazil Neuroendocrinology Group, Neurosurgery Division, Hospital das Clínicas, Faculdade de Medicina, Universidade de São Paulo, São Paulo, SP, Brazil.; 3 Universidade de São Paulo Faculdade de Medicina Instituto do Câncer do Estado de São Paulo São Paulo SP Brazil Division of Endocrine Oncology, Instituto do Câncer do Estado de São Paulo, Faculdade de Medicina, Universidade de São Paulo, São Paulo, SP, Brazil.

**Keywords:** Prolactinoma, Cabergoline, Pituitary neoplasms, Adenoma, Hyperprolactinemia

## Abstract

**Objective:**

Clinical, imaging, and laboratory differentiation between non-functioning pituitary adenomas (NFPAs) and prolactinomas can be challenging in clinical practice because of their similar presentation and eventual overlap in prolactin levels. The degree of prolactin suppression after a single low-dose cabergoline administration may be useful as a test to discriminate between these two clinical conditions and to determine the choice of therapy (dopamine agonist *versus* neurosurgery).

**Methods:**

This retrospective cohort study included 19 patients with macroadenomas (11 NFPAs and 8 prolactinomas) whose prolactin levels were evaluated at baseline and 48 h after receiving a single dose of 0.25mg cabergoline. A receiver operating characteristic curve was generated for sensitivity and specificity analyses.

**Results:**

The baseline median prolactin level was 58ng/mL (range, 32.5-151.2ng/mL) in the NFPA Group, which was significantly lower than that observed in the Prolactinoma Group (733.7 [range, 180-6941ng/mL]) (p<0.001). After the cabergoline disconnection test, the NFPA Group exhibited a lower absolute prolactin level (p<0.001) and a higher percentage of prolactin reduction (p=0.004). The post-test prolactin level ≥26.3ng/mL had 100% sensitivity and specificity in identifying prolactinomas. When using the percentage of reduction, post-test prolactin reduction ≥85.6% had 54.5% sensitivity and 100% specificity to differentiate NFPAs from prolactinomas.

**Conclusion:**

A single low-dose oral cabergoline is a fast and inexpensive test for use in daily clinical practice to discriminate between hyperprolactinemia due to NFPAs and prolactinomas, with high sensitivity and specificity.

## In Brief

Hyperprolactinemia due to prolactinoma is not always easy to differentiate from that caused by non-functioning pituitary adenomas. This pilot study revealed that low-dose (0.25mg) cabergoline could suppress prolactin levels within 48 h in non-functioning pituitary adenomas, helping to discriminate them from prolactinomas.

## Highlights

■ Hyperprolactinemia can be present in non-functioning pituitary adenomas because of the stalk effect.
■ Absolute prolactin levels may not always be reliable in discriminating non-functioning pituitary adenomas from prolactinomas.
■ Prolactin normalization after 48 h of 0.25mg cabergoline intake could be a strong predictor of non-functioning pituitary adenomas (100% sensitivity and specificity).

## Graphical abstract

**Figure:**
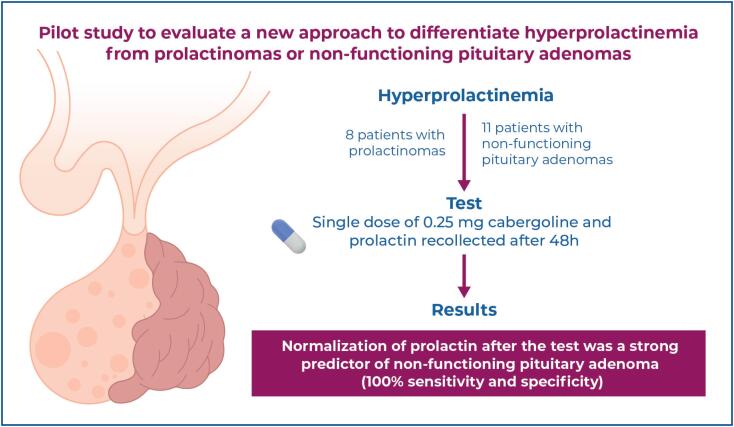

## INTRODUCTION

The first step in evaluating patients with elevated prolactin levels is to consider the possibility of autonomous prolactin secretion by a functioning pituitary tumor, usually a prolactinoma, which is the most frequent type of pituitary adenoma and the most common cause of pathological endogenous hyperprolactinemia. Before confirming the diagnosis, excluding secondary etiologies of hyperprolactinemia, such as physiological conditions ( *e.g* ., pregnancy, breastfeeding, stress, nipple stimulation), pharmacological agents ( *e.g* ., dopamine antagonists, antipsychotics, certain antidepressants), and systemic disorders ( *e.g* ., hypothyroidism, chronic kidney disease), is essential. Once these are ruled out based on clinical history, laboratory testing, and medication review, pituitary magnetic resonance imaging (MRI) should be performed to assess sellar or parasellar lesions. Even when a lesion is detected, other intrasellar etiologies such as non-functioning pituitary adenomas (NFPAs), craniopharyngiomas, meningiomas, and infiltrative diseases, which may cause hyperprolactinemia via the "stalk effect," should remain in the differential diagnosis.^( 1 )^

In the clinical investigation of hyperprolactinemia, differentiation between NFPAs and prolactinomas is crucial and has important therapeutic implications. For prolactinomas, dopamine agonists such as cabergoline are indicated as the first-line treatment to control prolactin levels and reduce tumor size. For NFPAs, neurosurgical treatment is the therapy of choice in cases with a "mass effect."^( 2 )^ Nevertheless, differential diagnosis can be challenging, especially when the prolactin levels are not elevated in the mid-range area (50-200ng/mL).^( 3 )^

**Figure 1:**
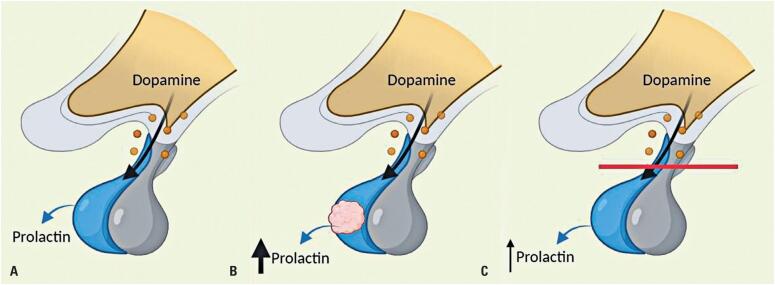
Mechanism of hyperprolactinemia in "stalk disconnection." (A) Physiological control of prolactin secretion: the dopaminergic tone inhibits normal lactotrophs, maintaining normal prolactin levels. (B) Autonomous prolactin secretion by a prolactinoma: prolactin production overrides physiologic dopamine inhibition, leading to variable elevations in prolactin levels. (C) Stalk compression or impairment ( *e.g* ., due to a large tumor or hypophysitis): this disrupts the dopaminergic tone, which normally inhibits lactotrophs, typically resulting in mild hyperprolactinemia

The prevalence of hyperprolactinemia in patients with NFPAs varies from 25% to 65%, with a mean prolactin level of 39 ng/mL (usually <100ng/mL).^( 4 , 5 )^ This increase in prolactin levels is justified by the mechanism known as the "stalk or disconnection effect" ( Figure 1 ), a result of a diminishing dopaminergic inhibition of lactotrophs when the pituitary stalk is compressed by large sellar tumors^( 6 )^ or affected by inflammatory/infectious conditions.^( 1 )^

Created with the BioRender tool. Available at: https://www.biorender.com/

Serum prolactin levels in macroprolactinomas (maximum tumor diameter, ≥1cm) usually exceed 250ng/mL, whereas those in microprolactinomas frequently range from 100 to 200ng/mL. However, serum prolactin levels <100ng/mL can occur in 25% of microprolactinomas. Although prolactin levels <100ng/mL in the presence of a macroadenoma suggest a diagnosis of NFPA,^( 7 )^ prolactin levels *per se* are not always sufficient to discriminate the cause of hyperprolactinemia owing to some overlapping values.^( 8 )^ Additionally, despite being rare, the "hook effect" can cause misdiagnosis between macroprolactinomas and NFPAs (very high prolactin concentrations lead to false-negative results).^( 9 )^

In our institution, patients with NFPAs have been empirically observed to present with fast normalization of prolactin levels a few days after cabergoline administration, whereas the response in patients with prolactinomas is generally slower and less pronounced. This observation could be explained by the uninhibited prolactin secretion at slightly elevated levels by normal lactotrophs present in the pituitary gland, with preserved sensitivity to dopamine agonists in NFPAs.

Considering that normal lactotrophs in patients with NFPAs are very sensitive to dopamine agonists, the use of low-dose cabergoline could completely resolve hyperprolactinemia and even suppress prolactin. Based on this, we explored whether a single dose of cabergoline could lower prolactin levels at higher rates in NFPAs than in prolactinomas and whether this would be a feasible test to differentiate between both conditions.

## OBJECTIVE

To evaluate whether low-dose (0.25mg) cabergoline could suppress prolactin levels in non-functioning pituitary adenomas and distinguish non-functioning pituitary adenomas from prolactinomas.

## METHODS

This pilot retrospective cohort study included 19 patients (11 with confirmed NFPAs and 8 with presumed or confirmed prolactinomas) between 2001 and 2019. Clinical, imaging, and laboratory parameters were collected from patients who underwent the cabergoline disconnection test at our institution, which is a tertiary center that focuses on neuroendocrine disorders.

All patients in the NFPA Group had macroadenomas, presented with hyperprolactinemia at baseline, and underwent neurosurgery; their diagnosis was confirmed through histopathological examination and hormonal immunohistochemistry analysis. Silent prolactinomas (presumed to be NFPA with positive prolactin staining by immunohistochemistry) were excluded. Patients in the Prolactinoma Group were included based on either a presumed or confirmed diagnosis. Presumed prolactinoma was defined as baseline serum prolactin levels exceeding 100ng/mL and tumor shrinkage ≥30% following cabergoline treatment. Prolactinomas were confirmed histologically and immunohistochemically in three cases: one due to cabergoline intolerance, one with tumor apoplexy, and one due to cerebrospinal fluid leakage. Hyperprolactinemia due to pregnancy, breastfeeding, macroprolactinemia, hypothyroidism, renal failure, cirrhosis, acromegaly, or drug use was ruled out.^( 10 )^

Prolactin was collected early in the morning using an indwelling peripheral venous catheter. Serum prolactin levels were measured using the Prolactin II electrochemiluminescence immunoassay (Cobas E601 and Cobas E602, Roche) with intra- and inter-assay variation coefficients of 0.8-1.7% and 1.4-2%, respectively. Routine prolactin dilution was performed to prevent the hook effect.^( 9 )^ The reference range for prolactin levels was 3.5-19.4 ng/mL in men and 5.2-26.5 ng/mL in women. Selective MRI was performed using scanners with a field strength of at least 1.5 Tesla. The examinations were carried out according to the usual protocol: 3mm multiplane images with T1, T2, and volumetric sequences. Coronal and sagittal images were acquired before and after intravenous gadolinium administration. Tumor volume was calculated using the classic formula validated by Lundin et al: 0.5 × width × length × height.^( 11 )^

The cabergoline disconnection test consisted of a single oral administration of 0.25mg cabergoline with prolactin measurement at baseline and 48 h after the test. The test was always performed during initial visits prior to definitive treatment (such as the introduction of a dopamine agonist or surgery). Notably, a minority of patients did not follow the aforementioned institutional protocol and inadvertently took a full tablet (0.5mg) of cabergoline, or a new prolactin was collected within the first week after the test (up to 168 h). The use of 48 h after cabergoline administration was based on classic studies regarding the pharmacokinetics and pharmacodynamics of cabergoline in healthy volunteers. When lactotrophs are normal, maximum prolactin suppression is usually observed within 48 h of low-dose cabergoline intake, and an increase is observed after more than 1 week.^( 12 )^

Data are summarized as means ± standard deviations or medians with ranges for continuous variables and as counts and percentages for categorical variables. The main outcomes evaluated were the absolute prolactin level after the test and the percentage of prolactin reduction in the two groups. The two groups were compared using the Mann-Whitney U and chi-square tests, and a receiver operating characteristic (ROC) curve was constructed for sensitivity and specificity analyses. Statistical analysis was performed using SPSS version 18.0, with statistical significance set at p<0.05.

This study was approved by the Research Ethics Committee of the *Hospital das Clínicas, Faculdade de Medicina, Universidade de São Paulo* (CAAE: 86439224.7.0000.0068; # 7.632.550). All participants signed an informed consent form.

## RESULTS

Nineteen patients were included: 11 (57.9%) with NFPAs and 8 (42.1%) with prolactinomas. The cohort consisted of 4 men (21%) and 15 women (79%), with a mean age of 47.3±13.2 years. All patients had macroadenomas. Additional clinical details are summarized in table 1 .

**Table 1:** Clinical and imaging characteristics of the included patients

	Case	Sex and age	Baseline prolactin	Prolactin (ng/mL) after 48 h of 0.25mg cabergoline (% of reduction)	Initial image (maximum diameter, aspect)	Treatment	Immunohistochemistry %	Volume reduction with cabergoline treatment %
**NFPAs**	1	Male, 66	36.2	10 (72)	2.5cm, solid	Surgery	FSH+ (>50), LH+ (10-50%)	-
	2	Female, 70	32.5	7.5 (77)	3.5cm, solid	Surgery	FSH+ (>50)	-
	3 †	Female, 41	36.9	3 (92)	4.0cm, solid	Surgery	TSH+ (<10), FSH+ (>50)	-
	4 †	Female, 58	45.3	0.5 (99)	1.0cm, solid	Surgery	Null cell	-
	5 †	Female, 36	58	11.8 (80)	2.5cm, solid	Surgery	Null cell	-
	6 †	Female, 63	65.4	1.2 (98)	2.0cm, solid	Surgery	Null cell	-
	7	Female, 43	61.2	23.6 (62)	1.5cm, solid	Surgery	Null cell	-
	8	Female, 48	101.9	7.1 (93)	3.1cm, solid	Surgery	FSH+ (<10), LH+ (<10)	-
	9	Female, 33	151.2	2.5 (98.3)	2.1cm, solid	Surgery	Null cell	-
	10	Female, 45	79	15 (81)	2.7cm, solid	Surgery	FSH+ (<10)	-
	11 ‡	Female, 37	42.9	5.5 (87.2)	2.4cm, solid	Surgery	FSH+ (10-50), LH+ (10-50)	-
**Prolactinomas**	1 ‡	Female, 36	199.8	98.9 (51)	1.1cm, cystic	0.5mg/w cabergoline	-	100
	2 ‡	Female, 37	180	29 (84)	1.4cm, solid	0.5mg/w cabergoline	-	68
	3	Female 27	527.1	169.9 (67.8)	1.7cm, solid	Surgery (cabergoline intolerance)	Prolactin+ (>50)	-
	4	Female, 35	6941	3238 (53.3)	4.5cm, solid	Surgery (apoplexy with visual loss)	Prolactin+ (>50)	-
	5	Female, 45	564.1	207.3 (63.2)	2.5cm, solid	0.5mg/w cabergoline	-	79
	6	Male, 60	1460	1122 (23.1)	2.2cm, solid	Surgery (cerebrospinal fluid leakage)	Prolactin+ (>50)	-
	7	Male, 69	5809	1299 (77.6)	4.3cm, solid	1.5mg/w cabergoline	-	97
	8	Male, 50	903.4	466.1 (50)	2.9cm, solid- cystic	0.5mg/w cabergoline	-	100

† Prolactin levels after 7 days (168 h);

‡ Intake of a full tablet (0.5mg) of cabergoline during the test.

NFPAs: non-functioning pituitary adenomas.

The mean age was similar between the Prolactinoma Group (44.8±14.1 years) and NFPA Group (47.2±13.4 years) (p=0.49). The sex distribution did not significantly differ between the groups (p=0.134). The NFPA Group had a median prolactin level of 58 ng/mL (range, 32.5-151.2ng/mL) at baseline, which was significantly lower than that observed in the Prolactinoma Group (733.7ng/mL [range, 180-6941ng/mL]) (p<0.001).

All patients in the NFPA Group exhibited normalized prolactin levels after cabergoline intake. After the cabergoline disconnection test, the mean prolactin level was 7.9±6.8ng/mL in the NFPA Group and 828.7±1084.6ng/mL in the Prolactinoma Group (p<0.001). The mean percentage of prolactin reduction was 85.4±12% in the NFPA Group, which was significantly higher than that in the Prolactinoma Group (58.7±19%) (p=0.004).

**Figure 2:**
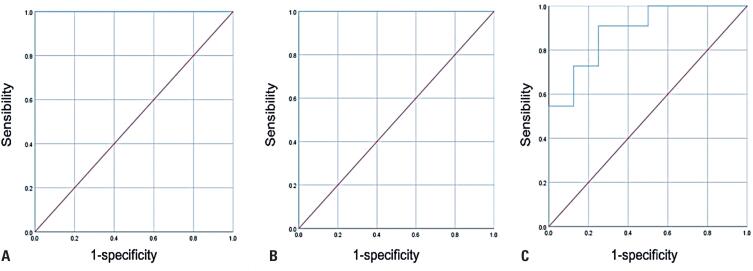
ROC curve for each parameter for the discrimination between non-functioning pituitary adenomas and prolactinomas. (A) ROC curve for the absolute baseline prolactin value. (B) ROC curve for the absolute post-test prolactin value. (C) ROC curve for the post-test percentage of prolactin reduction

Upon evaluation, baseline prolactin level ≥165.6ng/mL showed 100% sensitivity and specificity for prolactinomas, with an area under the ROC curve (AUC) of 1 ( Figure 2A ). Similarly, post-test prolactin level ≥26.3ng/mL (upper limit of reference for our method) exhibited 100% sensitivity and specificity in identifying prolactinomas, with an AUC of 1 ( Figure 2B ).

When using the percentage of reduction, post-test prolactin reduction ≥85.6% showed 54.5% sensitivity and 100% specificity to discriminate NFPAs from prolactinomas, with an AUC of 0.886 (95% confidence interval: 0.737-1) ( Figure 2C ).

## DISCUSSION

Several strategies have been proposed to help clinicians differentiate NFPAs from prolactinomas. The first approach proposed in the literature is the use of baseline prolactin values. However, every center presents different cut-off values, ranging from 40 to 150ng/mL,^( 3 , 13 , 14 )^ accompanied by variable sensitivity and specificity rates. According to the 2023 Pituitary Consensus, prolactin level ≥200ng/mL is more suggestive of prolactinomas.^( 1 )^ Nonetheless, as previously mentioned, prolactin levels are overlapping in both conditions. Some clinical characteristics such as old age, extrasellar tumor extension with relatively low prolactin levels, visual defects, and growth hormone (GH) deficiency favor NFPAs. By contrast, galactorrhea and amenorrhea/hypogonadotropic hypogonadism are suggestive of prolactinomas.^( 15 )^

Another approach is to evaluate the ratio between prolactin levels and tumor volume or dimensions ( Table 2 ). It is based on the proportionality of prolactin levels and tumor mass in prolactinomas, which is not linear in NFPAs. Nevertheless, this strategy is not ideal for patients harboring cystic prolactinomas, in which prolactin levels can vary from 50 to 150ng/mL^( 1 )^ and are directly correlated with the smaller solid mass of hyperfunctioning lactotrophs.

**Table 2:** Clinical studies proposing predictors to differentiate non-functioning pituitary adenomas from prolactinomas

Author and year	Patients	Parameters used	Comments
Present study	19 (11 NFPAs and 8 prolactinomas)	Prolactin value after 48 h on 0.25mg cabergoline	A post-test prolactin level of 26.3ng/mL had an AUC of 1 (the best reported in the literature to date)
Kim et al., 2022^( 8 )^	223 (175 NFPAs and 48 prolactinomas)	Ratio of serum prolactin and maximal tumor diameter	A cut-off value of 8.93 (ug/L)/mm had an AUC of 0.938
Cho et al., 2022^( 25 )^	55 (39 prolactinomas and 11 other sellar lesionsº)	Prolactin-to-volume ratio	The Prolactinoma Group was younger and had higher prolactin levels, small tumor volume, and lower degree of stalk deviation; prolactin-to-volume ratio >100ng/mL percm^3^ had an AUC of 0.77
Wright et al., 2021^( 26 )^	79 (58 NFPAs and 21 prolactinomas)	Prolactin-to-tumor volume ratio	An optimal cut-off value of 21.62 (ng/mL)/cm^3^ had an AUC of 0.9647
Burke et al., 2019^( 27 )^	293 (76 prolactinomas and 217 NFPAs)	Absolute prolactin value stratified by lesion volume	Several cut-off values and AUC of prolactin value depending on the initial adenoma volume (<0.5, 0.5-4, >4cm^3^); prolactin levels in NFPAs did not correlate well with the tumor mass volume
Huang et al., 2018^( 28 )^	118 (30 prolactinomas and 88 other pituitary adenomas)	Prolactin-to-tumor volume ratio	A cut-off point of 54.0 (ng/mL)/cm^3^ had an AUC of 0.947

These included NFPAs (n=7), Rathke's cyst (n=7), hypophysitis (n=1), and intrasellar meningioma (n=1).

NFPAs: non-functioning pituitary adenomas; BRC: bromocriptine; AUC: area under the curve.

Importantly, tumor volume shrinkage ≥30% in macroprolactinomas can be noticed during the first 3-4 months of cabergoline therapy.^( 16 )^ Therefore, in patients without significant visual impairment, when clinical observation is feasible, a therapeutic test with cabergoline can be used to aid in differentiating prolactinomas from NFPAs. However, patients with NFPAs on cabergoline may have tumor shrinkage.^( 17 )^ Although this is usually minimal (0.5mm), tumor shrinkage could reach approximately 20% (5mm) in good responders within the first years of treatment,^( 18 )^ rendering its distinction from resistant prolactinomas troublesome. In addition, prolactinomas may show a significant drop in prolactin levels, independent of a proportional reduction in tumor size.^( 19 )^ Unfortunately, this strategy is not applicable to microadenomas.

With regard to MRI for the analysis of stalks in NFPAs, the data are controversial. A previous study reported no significant correlation between prolactin levels and the degree of pituitary stalk compression, stalk deviation, or tumor size. Prolactin levels were markedly elevated in some patients with tumors, causing minimal distortion of the pituitary stalk; conversely, prolactin levels were often normal despite evidence of massive stalk distortion.^( 20 )^ A recent study involving 107 patients with NFPAs demonstrated that the presence of a cystic or hemorrhagic tumor and the presence of pituitary stalk deviation were statistically more frequent in patients with hyperprolactinemia. Surprisingly, the incidence of postoperative transient diabetes insipidus was also statistically significantly higher in the group with prolactin levels ≥40 ng/mL and in the group with radiologic evidence of stalk deviation.^( 6 )^

Rarely, non-functioning microadenomas causing hyperprolactinemia have been described, with increased intrasellar pressure without apparent stalk deviation as the possible mechanism.^( 21 )^ In our study, the smallest NFPA presenting with hyperprolactinemia had a maximal dimension of 1.0cm. Although our pilot project included patients with a somewhat clear diagnosis ( *i.e* ., large NFPA with prolactin levels <100ng/mL and macroprolactinoma with prolactin levels >500ng/mL), this was intended to serve as a conceptual proof for the rationale of the test. In the present study, a few patients (cases 1 and 2 in the Prolactinoma Group and cases 8 and 9 in the NFPA Group) posed a diagnostic dilemma. For these patients, the cabergoline disconnection test would add information because the post-test prolactin level in the two cases of NFPAs was within the normal range (in fact, next to the lower limit of normality).

The inclusion of cystic lesions in the Prolactinoma Group also expanded the usefulness of the cabergoline disconnection test. As the behavior after cabergoline administration in this situation did not change (maintained the attenuated reduction in prolactin values when compared to NFPAs), this is an advantage when compared to the volume parameters previously reported in the literature. The cabergoline test can also be used in resistant prolactinoma cases with the same rationale.

Radiomics has recently been proposed as a method for identifying prolactinomas and differentiating them from other sellar tumors using multiparametric MRI.^( 22 )^ Radiomics is a quantitative approach to medical image analysis that aims to decode the morphological and functional characteristics of a lesion and correlate them with histology, prognosis, and therapeutic response.^( 23 )^ Nonetheless, challenges such as the lack of standardization in radiomic feature extraction (dynamic *versus* static),^( 24 )^ the need for advanced machine learning algorithms, and complex statistical validation remain significant hurdles.^( 23 )^ Further studies are required to establish the applicability of this technique in routine clinical practice.

This study had some limitations. The sample size was small, and not all patients in the Prolactinoma Group underwent neurosurgery for histological confirmation. Additionally, a minority of patients did not fully adhere to the study protocol, either receiving a full tablet (0.5mg) of cabergoline or failing to undergo new prolactin measurements within 48 h. Despite these deviations, the prolactin reduction patterns remained similar. Furthermore, the presumed or confirmed Prolactinoma Group included patients with extremely high prolactin levels (up to 6941ng/mL), which might have introduced a "performance" bias, potentially resulting in a smaller prolactin reduction when compared to prolactinomas with lower baseline prolactin levels.

In the future, we plan to expand the test to a larger cohort in a prospective trial to further evaluate its applicability in daily clinical practice, with a particular focus on borderline cases (typically with prolactin levels between 100 and 200ng/mL). In honor of Dr. Marcello Delano Bronstein, the former head of the Neuroendocrinology Unit in our department and the visionary behind this test, we propose naming it "Bronstein's test."

## CONCLUSION

The cabergoline disconnection test is a simple, fast, inexpensive, and useful tool for differentiating non-functioning pituitary adenomas from prolactinomas with high sensitivity and specificity. We suggest that, in cases of macroadenomas, normalization of prolactin levels (prolactin levels <26.3ng/mL) after cabergoline administration should be considered a strong predictor of non-functioning pituitary adenomas.

DATA AVAILABILITY STATEMENT

The data supporting the findings of this study are available within the article and can be obtained from the corresponding author upon reasonable request.

## References

[1] Petersenn S, Fleseriu M, Casanueva FF, Giustina A, Biermasz N, Biller BM (2023). Diagnosis and management of prolactin-secreting pituitary adenomas: a Pituitary Society international Consensus Statement. Nat Rev Endocrinol.

[2] Glezer A, Bronstein MD (2015). Prolactinomas. Endocrinol Metab Clin North Am.

[3] Vilar L, Freitas MC, Naves LA, Casulari LA, Azevedo M, Montenegro R Jr (2008). Diagnosis and management of hyperprolactinemia: results of a Brazilian multicenter study with 1234 patients. J Endocrinol Invest.

[4] Fleseriu M, Bodach ME, Tumialan LM, Bonert V, Oyesiku NM, Patil CG (2016). Congress of neurological surgeons systematic review and evidence-based guideline for pretreatment endocrine evaluation of patients with nonfunctioning pituitary adenomas. Neurosurgery.

[5] Zhang F, Huang Y, Ding C, Huang G, Wang S (2015). The prevalence of hyperprolactinemia in non-functioning pituitary macroadenomas. Int J Clin Exp Med.

[6] Sakata K, Hashimoto A, Takeshige N, Orito K, Nagayama A, Ashida K (2024). Clinical and radiographic characteristics of patients with non-functioning pituitary adenomas categorized according to their serum prolactin concentration: novel predictors of postoperative transient diabetes insipidus following surgery. Endocrine.

[7] Karavitaki N, Thanabalasingham G, Shore HC, Trifanescu R, Ansorge O, Meston N (2006). Do the limits of serum prolactin in disconnection hyperprolactinaemia need re-definition? A study of 226 patients with histologically verified non-functioning pituitary macroadenoma. Clin Endocrinol (Oxf).

[8] Kim JH, Hur KY, Hong SD, Choi JW, Seol HJ, Nam DH (2022). Serum prolactin level to tumor size ratio as a potential parameter for preoperative differentiation of prolactinomas from hyperprolactinemia-causing non-functional pituitary adenomas. World Neurosurg.

[9] Raverot V, Perrin P, Chanson P, Jouanneau E, Brue T, Raverot G (2022). Prolactin immunoassay: does the high-dose hook effect still exist?. Pituitary.

[10] Glezer A, Mendes Garmes H, Kasuki L, Martins M, Condé Lamparelli Elias P, Dos Santos Nunes Nogueira V (2024). Diagnosis of hyperprolactinemia in women: A Position Statement from the Brazilian Federation of Gynecology and Obstetrics Associations (Febrasgo) and the Brazilian Society of Endocrinology and Metabolism (SBEM). Arch Endocrinol Metab.

[11] Lundin P, Pedersen F (1992). Volume of pituitary macroadenomas: assessment by MRI. J Comput Assist Tomogr.

[12] Andreotti AC, Pianezzola E, Persiani S, Pacciarini MA, Strolin Benedetti M, Pontiroli AE (1995). Pharmacokinetics, pharmacodynamics, and tolerability of cabergoline, a prolactin-lowering drug, after administration of increasing oral doses (0.5, 1.0, and 1.5 milligrams) in healthy male volunteers. J Clin Endocrinol Metab.

[13] Kawaguchi T, Ogawa Y, Tominaga T (2014). Diagnostic pitfalls of hyperprolactinemia: the importance of sequential pituitary imaging. BMC Res Notes.

[14] Bevan JS, Burke CW, Esiri MM, Adams CB (1987). Misinterpretation of prolactin levels leading to management errors in patients with sellar enlargement. Am J Med.

[15] Hong JW, Lee MK, Kim SH, Lee EJ (2010). Discrimination of prolactinoma from hyperprolactinemic non-functioning adenoma. Endocrine.

[16] Biagetti B, Sarria-Estrada S, Ng-Wong YK, Martinez-Saez E, Casteràs A, Cordero Asanza E (2021). Shrinkage by the third month predicts long-term response of macroprolactinoma after cabergoline. Eur J Endocrinol.

[17] Batista RL, Musolino NR, Cescato VA, da Silva GO, Medeiros RS, Herkenhoff CG (2019). Cabergoline in the management of residual nonfunctioning pituitary adenoma: a single-center, open-label, 2-year randomized clinical trial. Am J Clin Oncol.

[18] Ayalon-Dangur I, Turjeman A, Hirsch D, Robenshtok E, Tsvetov G, Gorshtein A (2024). Cabergoline treatment for surgery-naïve non-functioning pituitary macroadenomas. Pituitary.

[19] Molitch ME (2008). Nonfunctioning pituitary tumors and pituitary incidentalomas. Endocrinol Metab Clin North Am.

[20] Smith MV, Laws ER (1994). Magnetic resonance imaging measurements of pituitary stalk compression and deviation in patients with nonprolactin-secreting intrasellar and parasellar tumors: lack of correlation with serum prolactin levels. Neurosurgery.

[21] Simander G, Dahlqvist P, Oja L, Eriksson PO, Lindvall P, Koskinen LD (2023). Intrasellar pressure is related to endocrine disturbances in patients with pituitary tumors. World Neurosurg.

[22] Li H, Liu Z, Li F, Xia Y, Zhang T, Shi F (2024). Identification of prolactinoma in pituitary neuroendocrine tumors using radiomics analysis based on multiparameter MRI. J Imaging Inform Med.

[23] Shaikh FA, Kolowitz BJ, Awan O, Aerts HJ, von Reden A, Halabi S (2017). Technical challenges in the clinical application of radiomics. JCO Clin Cancer Inform.

[24] van Griethuysen JJ, Fedorov A, Parmar C, Hosny A, Aucoin N, Narayan V (2017). Computational radiomics system to decode the radiographic phenotype. Cancer Res.

[25] Cho A, Vila G, Marik W, Klotz S, Wolfsberger S, Micko A (2022). Diagnostic criteria of small sellar lesions with hyperprolactinemia: prolactinoma or else. Front Endocrinol (Lausanne).

[26] Wright K, Lee M, Escobar N, Pacione D, Young M, Fatterpekar G (2021). Tumor volume improves preoperative differentiation of prolactinomas and nonfunctioning pituitary adenomas. Endocrine.

[27] Burke WT, Penn DL, Castlen JP, Donoho DA, Repetti CS, Iuliano S (2019). Prolactinomas and nonfunctioning adenomas: preoperative diagnosis of tumor type using serum prolactin and tumor size. J Neurosurg.

[28] Huang Y, Ding C, Zhang F, Xiao D, Zhao L, Wang S (2018). Role of prolactin/adenoma maximum diameter and prolactin/adenoma volume in the differential diagnosis of prolactinomas and other types of pituitary adenomas. Oncol Lett.

